# Oncogenic Viruses and the Epigenome: How Viruses Hijack Epigenetic Mechanisms to Drive Cancer

**DOI:** 10.3390/ijms24119543

**Published:** 2023-05-31

**Authors:** Signe A. MacLennan, Marco A. Marra

**Affiliations:** 1 Department of Medical Genetics, University of British Columbia, Vancouver, BC V6T 1Z4, Canada; 2 Michael Smith Laboratories, University of British Columbia, Vancouver, BC V6T 1Z4, Canada; 3 Canada’s Michael Smith Genome Sciences Centre, BC Cancer, Vancouver, BC V5Z 4S6, Canada

**Keywords:** oncogenic viruses, epigenome dysregulation, viral oncoproteins, DNA methylation, histone post-translational modifications, non-coding RNAs, histone deacetylase inhibitors

## Abstract

Globally, viral infections substantially contribute to cancer development. Oncogenic viruses are taxonomically heterogeneous and drive cancers using diverse strategies, including epigenomic dysregulation. Here, we discuss how oncogenic viruses disrupt epigenetic homeostasis to drive cancer and focus on how virally mediated dysregulation of host and viral epigenomes impacts the hallmarks of cancer. To illustrate the relationship between epigenetics and viral life cycles, we describe how epigenetic changes facilitate the human papillomavirus (HPV) life cycle and how changes to this process can spur malignancy. We also highlight the clinical impact of virally mediated epigenetic changes on cancer diagnosis, prognosis, and treatment.

## 1. Introduction

Despite public health measures, including vaccines, oncogenic viruses remain important drivers relevant to multiple cancer types [1,2]. Virally driven cancers represent ~13–20% of all cancers globally [1,3] and ~45% of cancers in parts of sub-Saharan Africa [2]. Even in regions with historically low rates of virally driven cancers [2], oncogenic viruses remain relevant, as exemplified by the rising incidence of human papillomavirus (HPV)-driven head and neck squamous cell carcinomas (HNSCCs) in high-income countries [4].

**Figure 1:**
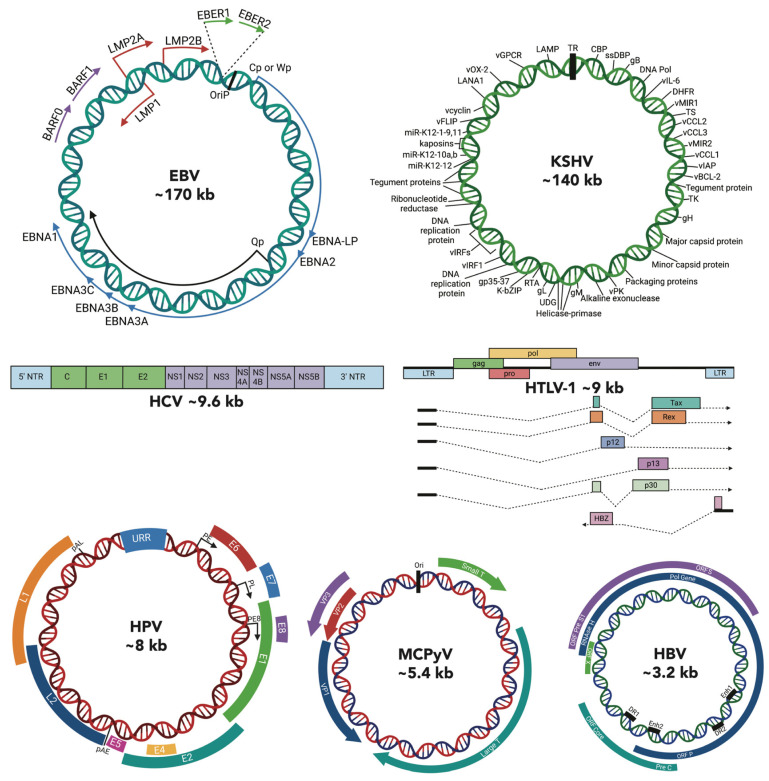
Oncogenic viruses: genomic structures. Genomic structures of each of the seven established human oncogenic viruses, ordered from largest to smallest (not to scale). Note that only one virus type is shown per oncogenic virus, but the position of genes does vary slightly between different types. BARF0/1—BamHI A right frame 0/1; LMP1/2A/2B—latent membrane protein 1/2A/2B; EBER1/2—EBV-encoded small RNAs 1/2; EBNA1/2/3A/3B/3C—EBV nuclear antigen 1/2/3A/3B/3C; EBNA-LP—EBV nuclear antigen-leader protein; Cp—C promoter; Wp—W promoter; OriP—origin of plasmid replication; TR—terminal repeat; CBP—complement-binding protein; ssDBP—single-stranded DNA binding protein; gB/M/H—glycoprotein B/M/H; DNA Pol—DNA polymerase; vIL-6—viral interleukin-6; DHFR—dihydrofolate reductase; vMIR1/2—modulator of immune recognition 1/2; TS—thymidylate synthase; vCCL1/2/3—viral CC-chemokine ligand 1/2/3; vBCL-2—viral B-cell leukaemia-2; TK—thymidine kinase; vPK—viral protein kinase; UDG—uracil DNA-glycosylase; RTA—replication and transcription activator; k-bZIP—KSHV basic region-leucine zipper protein; gp35-37—glycoprotein35-37; vIRF1—viral interferon regulatory factor 1; miR—microRNA; vFLIP—viral Fas-associated death domain-like interleukin-1β-converting enzyme inhibitory protein; LANA—latency-associated nuclear antigen; vcyclin—viral cyclin; vOX-2—viral OX2; vGPCR—viral G-protein coupled receptor; LAMP—latency-associated membrane protein; NTR—non-translated region; NS1/2/3/4A/5A/5B—non-structural protein 1/2/3/4A/5A/5B; LTR—long terminal repeat; HBZ—HTLV-1 bZIP (basic region leucine-zipper) factor; URR—upstream regulatory region; PE—early promoter; PL—late promoter; pAL—late polyadenylation site; pAE—early polyadenylation site; VP1/2/3—viral protein 1/2/3; ORF—open reading frame; DR1/2—direct repeat 1/2; Enh1/2—enhancer 1/2. Adapted from [21,22,23,24,25,26,27]. Created with BioRender.com (accessed on 28 May 2023).

Evidence for the link between viruses and cancer was first uncovered more than one century ago when Dr. Peyton Rous famously discovered that, upon filtration to remove tumour cells and bacteria, extracts from chicken fibrosarcoma were transmissible [5]. Subsequently, the association between Epstein–Barr virus (EBV) and Burkitt’s lymphoma extended the role of viruses to human cancer [6]. As recognized by the World Health Organization (WHO), there are now seven human oncogenic viruses: EBV, high-risk HPVs (e.g., HPV16 and HPV18), hepatitis B virus (HBV), hepatitis C virus (HCV), human T-lymphotropic virus-1 (HTLV-1), Kaposi sarcoma herpesvirus/human herpesvirus 8 (KSHV/HHV-8), and Merkel cell polyomavirus (MCPyV), which are linked to nearly 20 different malignancies (Table 1, Figure 1) [7].

Oncogenic viruses are taxonomically diverse and exhibit a broad range of tissue tropisms [7]. Oncogenic viruses dysregulate a wide variety of host-cell oncogenic pathways, resulting in loss of cell cycle control, inhibition of apoptosis, and immune evasion [1]. For example, the majority of oncogenic viruses produce oncoproteins that converge on pRB and p53, ultimately fueling transformation to malignancy by preventing cell cycle arrest and apoptosis [1]. More recently, our understanding of how oncogenic viruses drive cancer has expanded to include epigenetic mechanisms [8].

**Table 1:** Oncogenic viruses and their associated malignancies.

Oncogenic Virus	Genome	Family	Associated Cancer Types	Global Infection Prevalence	Global Attributable Fraction	Refs.
EBV	dsDNA ~170 kb	Herpesviridae	− BL − HL − ENKTL − DLBCL − NPC − GC − Paediatric − LMS	− >90%	− BL (~55%) − HL (~46–58%) − ENKTL (100%) − DLBCL (~4–13%) − NPC (~85%) − GC (~8–10%) − Paediatric LMS (LD) − All cancers (~1.5%)	[3,9]
HPVs	dsDNA ~8 kb	Papillomaviridae	− CC − HNSCC − AC − EV-associated	− ~75%	− CC (>95%) − HNSCC (~30% oropharyngeal, ~2% oral, ~2% laryngeal) − AC (anal ~88%, vulvar ~25%, vaginal ~78%, penile ~50%) − EV-associated (LD) − All cancers (~4.5%)	[3,10,11]
HBV	dsDNA ~3.2 kb	Hepadnaviridae	− HCC	− ~4%	− HCC (~56%)	[12,13]
HCV	ssRNA ~9.6 kb	Flaviviridae	− HCC − NHL	− ~1%	− HCC (~20%) − NHL (~3%)	[13,14]
HTLV-1	ssRNA ~9 kb	Retroviridae	− ATL	− <1%	− ATL (100%)	[3,15]
KSHV	dsDNA ~165 kb	Herpesviridae	− KS − NHL	− <10%	− KS (100%) − NHL (LD)	[2,3]
MCPyV	dsDNA ~5.4 kb	Polyomaviridae	− MCC	− ~80%	− MCC (~36–80%)	[16]

EBV—Epstein–Barr virus; HPV—human papillomavirus; HBV—hepatitis B virus; HCV—hepatitis C virus; HTLV-1—human T cell leukaemia virus type 1; KSHV—Kaposi’s sarcoma-associated herpesvirus; MCPyV—Merkel cell polyomavirus; ds—double-stranded; ss—single-stranded; kb—kilobase pairs; BL—Burkitt’s lymphoma; HL—Hodgkin’s lymphoma; ENKTL—extranodal natural killer/T cell lymphoma; DLBCL—diffuse large B cell lymphoma; NPC—nasopharyngeal carcinoma; GC—gastric carcinoma; LMS—leiomyosarcoma; CC—cervical cancer; HNSCC—head and neck squamous cell carcinoma; AC—anogenital carcinoma; EV—epidermodysplasia verruciformis; HCC—hepatocellular carcinoma; ATL—adult T cell leukaemia/lymphoma; KS—Kaposi’s sarcoma; NHL—non-Hodgkin’s lymphoma; MCC—Merkel cell carcinoma; LD—lacking data.

The term epigenetics encompasses chromatin modifications and mechanisms that impact gene regulation without changing the DNA sequence itself [17]. The growing list of such mechanisms includes, but is not limited to, DNA methylation, histone post-translational modifications (PTMs), and non-coding RNAs (ncRNAs). Epigenetic modifications can result in the widespread dysregulation of multiple genes, including those with ties to established cancer hallmarks [17,18,19]. Epigenetic dysregulation in cancer is now recognized as a key contributor to malignancy, and the plasticity of the epigenome has implications for targeted therapy development (reviewed in [20]). Thus, uncovering oncogenic epigenetic mechanisms in cancer has been, and continues to be, vital to the goal of developing better treatments and, ideally, cures.

Oncogenic viruses utilize host epigenetic machinery and modify host epigenomes, thus contributing to carcinogenesis [28,29,30]. Viruses utilize host epigenetic modifiers to regulate viral gene expression [28], segregate their genomes into daughter cells [29], and maintain viral latency to evade the host’s immune system [28,30]. From the evolutionary perspective of the virus, cancer is not a goal, but rather a side-effect of such hijacking [31]. Indeed, by driving cancer initiation, oncogenic viruses most often lose the ability to infect new hosts and thus suffer a significant loss of fitness [31].

Thus, studying viral mechanisms modulating host epigenomes is important to both the oncology and virology fields. This review aims to highlight the consequences of virally mediated epigenetic changes on cancer pathophysiology. We will focus specifically on the virally driven epigenetic mechanisms that promote cancer development, with brief mention of how such mechanisms contribute to the maintenance of oncogenic viral life cycles. The potential prognostic, diagnostic, and therapeutic utility of virally associated epigenetic marks in cancer will also be discussed. Finally, we will conclude with a prediction as to where this exciting multi-disciplinary field is heading.

## 2. Cancer Epigenetics

### 2.1. DNA Methylation

The methylation status of CpG sites in the genome has long been recognized for its regulatory role in gene transcription and many other cellular processes [32]. Methylation patterns are frequently disrupted in malignancy, at both global and gene-level scales. Global hypomethylation is a common feature of many cancers [32], and the CpG island methylator phenotype (CIMP) has been well-described in IDH-mutated low-grade glioma [33,34], colorectal cancer [35], and gastric carcinoma (GC) [36]. At the gene level, promoter hypermethylation and subsequent reduced gene expression has been observed in multiple tumour suppressor genes with roles in cell cycle control (e.g., *CDKN2A* and *CDKN2B*), adhesion (e.g., *CDH1*), and DNA repair (e.g., *MLH1* and *BRCA1*) [32]. In cancer, DNA methylation also serves as a predictive biomarker, as exemplified by *MGMT* promoter hypermethylation and its association with response to temozolomide in a subset of brain tumours [37]. DNA methylation of viral genomes is intricately choreographed and allows oncogenic viruses to progress through their life cycles [38,39,40]. For example, the co-option of host DNA methyltransferases (DNMTs) allows oncogenic viruses to switch between latent and lytic cycles [40]. Viral infection can also more directly result in host genome dysregulation by inducing host DNA methylation changes [41,42,43].

### 2.2. Histone Post-Translational Modifications

Chromatin is composed of a series of DNA-protein complexes that play roles in DNA compaction and regulation of gene expression [18]. The fundamental unit of chromatin, the nucleosome, consists of ~147 bp of DNA wrapped around a histone octamer that is composed of heterodimers of proteins H2A, H2B, H3, and H4, which are collectively referred to as core histone subunits. PTMs of these core histones at key residues alter how tightly DNA is associated with the histones and consequently dictate how accessible gene promoters and enhancers are to transcriptional machinery [18]. For example, lysine acetylation (e.g., histone 3 at lysine 27, abbreviated as H3K27ac) neutralizes a positive charge and leads to a loosening of the association with the negatively charged DNA backbone, thereby opening up the chromatin and allowing for binding of transcription factors (TFs) to promote gene transcription [44]. There are many layers of regulation at the level of histone PTMs, as effects on gene transcription depend both on the position of the residue and the nature of the chromatin modification. The “histone code” is established, modified, or read by protein complexes that add (i.e., “writers”), interpret (i.e., “readers”), or remove (i.e., “erasers”) these marks [18]. Errors in any stage of chromatin remodelling can contribute to malignancy, including errors resulting from viral infection [45].

### 2.3. Non-Coding RNAs

ncRNAs are non-translated gene products, such as microRNAs (miRNAs), long non-coding RNAs (lncRNAs), circular RNAs (circRNAs), and small nuclear RNAs (snRNAs), as well as key players in protein translation, transfer RNAs (tRNAs), and ribosomal RNAs (rRNAs) [46]. Notably, miRNAs play an important role in post-translational gene expression regulation. By binding to the 3′ untranslated region (UTR) of different mRNA transcripts through recognition of similar binding site sequences, a single miRNA molecule can inhibit the expression of multiple genes. In a highly context-dependent manner, miRNAs can act as either tumour suppressors or as oncogenes in cancer [46]. Different ncRNA classes also interact with each other, as exemplified by circRNAs acting as miRNA sponges in order to mute miRNA expression [47]. The ncRNAs with the most well-established roles in cancer are miRNAs, lncRNAs, and circRNAs, for all of which there is evidence of interactions with oncogenic viruses [48,49,50,51].

## 3. Epigenetics and Viral Life Cycles: HPV in Cervical Cancer as a Case Study

For transmission to new hosts, HPV and other oncogenic viruses must generate new virion progeny through productive infection [25,52]. Yet, if HPV is not cleared by the immune system, it may cease active replication of its genome as an unwelcome persistent passenger within host cells. In this persistent infection state, HPV can promote oncogenesis via multiple mechanisms, including by the action of viral oncoproteins. Thus, cancer is a consequence of HPV diverging from its normal life-cycle trajectory [25]. Here, we provide an example of how an oncogenic virus’s life cycle can go awry and lead to malignancy. We describe differences between productive and persistent infection, illustrate how epigenetic hijacking is utilized by HPV in various stages of its life cycle, and highlight how each epigenetic mechanism ultimately contributes to cervical cancer development.

### 3.1. The HPV Life Cycle—Productive Infection vs. Neoplastic Progression

The HPV life cycle, including the regulation of the viral epigenome, is intimately tied to the differentiation status of the host cell [25]. Through a microlesion, HPV infects undifferentiated basal keratinocytes of a stratified squamous epithelium. Following infection, HPV replicates its episomal genome ~50–100 times using the host cell’s transcriptional machinery. HPV transcribes its viral genes through an early and a late promoter, which govern the expression of viral oncogenes to coincide with the migration of the host cell towards the uppermost layers of the epithelium [25]. As their names suggest, activation of the early promoter results in expression of the early genes (*E1*, *E2*, *E5*, *E6*, and *E7*), whereas activation of the late promoter occurs in terminally differentiated keratinocytes and results in expression of the viral capsid genes, *L1* and *L2*, as well as *E4* [25]. Ultimately, the orchestration of the productive HPV life cycle results in virion progeny being released to infect new host cells. Most HPV infections are cleared by the immune system. However, if HPV infection becomes persistent, viral oncoproteins can contribute to cancer formation alongside additional genetic and epigenetic alterations [25].

### 3.2. Epigenetic Modulation of HPV and Host Gene Expression in Cervical Cancer

HPV epigenome changes and interactions with chromatin modifiers occur as part of the HPV life cycle [25,29]. For example, HPV E2 interacts with host BRD4, a host chromatin-modifying enzyme, to attach replicated viral episomes to host chromatin in order to evenly partition the episomes into daughter cells [29]. Epigenetic changes to the HPV genome can lead to the constitutive expression of the viral oncogenes *E6* and *E7*, both of which have multiple roles in cancer initiation and progression [53,54]. For instance, E2 is a negative regulator of both *E6* and *E7* expression. When *E2* expression is disrupted (e.g., via viral integration into the host genome), *E6* and *E7* expression and activity increase [55,56]. The E2 binding sites within the upstream regulatory region facilitate E2′s repressive action, and when CpGs within this region are methylated, E2 binding is physically blocked, promoting viral oncogene expression [57]. Generally, in productive infection, E2 binding sites tend to be hypermethylated in undifferentiated host cells but become hypomethylated as host cells transition to more differentiated states, coinciding with increased E2 function [57]. Yet, in cervical cancer initiation, *E2* expression is continually blocked, allowing *E6* and *E7* expression to be maintained without negative regulation [25].

In the progression from pre-cancerous lesions to high-grade cervical cancer, many epigenetic changes occur in both the HPV and the host genome [58,59]. In general, the HPV genome is highly methylated in cervical cancer. Specific methylation differences can distinguish between non-cancerous tissues and different grades of lesions, indicating regulation of the HPV methylome throughout neoplastic progression [58,59]. The openness of HPV’s chromatin also varies in accordance with keratinocyte differentiation, and this coordinates HPV gene expression programmes [60]. Furthermore, both E6 [61,62] and E7 [63] can inhibit p300, the histone acetyltransferase (HAT) enzyme, thereby limiting p300′s E2 transactivation activity and further promoting *E6* and *E7* expression by a positive feedback loop. Predictably, host cells have evolved defensive mechanisms to prevent *E6* and *E7* transcription. One such mechanism is via the TF Yin Yang 1 (YY1), which represses *E6* and *E7* expression [64,65] by blocking access to their enhancer with CTCF-dependent looping of HPV chromatin [66]. As keratinocytes differentiate, *YY1* expression is lost, which alleviates this repressive loop and allows for *E6* and *E7* to be transcribed [66]. In summary, there are now numerous studies providing evidence that epigenetic regulation varies widely between the productive HPV life cycle and the formation of cervical cancer.

## 4. Impacts of Virally Mediated Epigenetic Changes on Cancer Pathology

Here, we discuss mechanisms by which oncogenic viruses hijack host epigenetic machinery and modify host epigenomes to drive malignancy. Examples of virally mediated epigenetic changes that have clear links to cancer pathology are highlighted in Table 2. 

### 4.1. Epithelial-to-Mesenchymal Transition

The epithelial-to-mesenchymal transition (EMT) describes the dynamic process in which epithelial cells lose cell adhesion and adopt a more motile mesenchymal phenotype through a coordinated pattern of gene expression changes orchestrated by transcription factors (TFs) from the TWIST, SNAIL, and ZEB families [85]. In cancer, EMT is intimately linked to metastatic potential. EMT is now recognized to be mediated by a spectrum of gene expression changes and is not considered to be a binary switch in cell phenotype [86]. EMT is a fundamental process in cancer and is exploited by oncogenic viruses.

**Figure 2:**
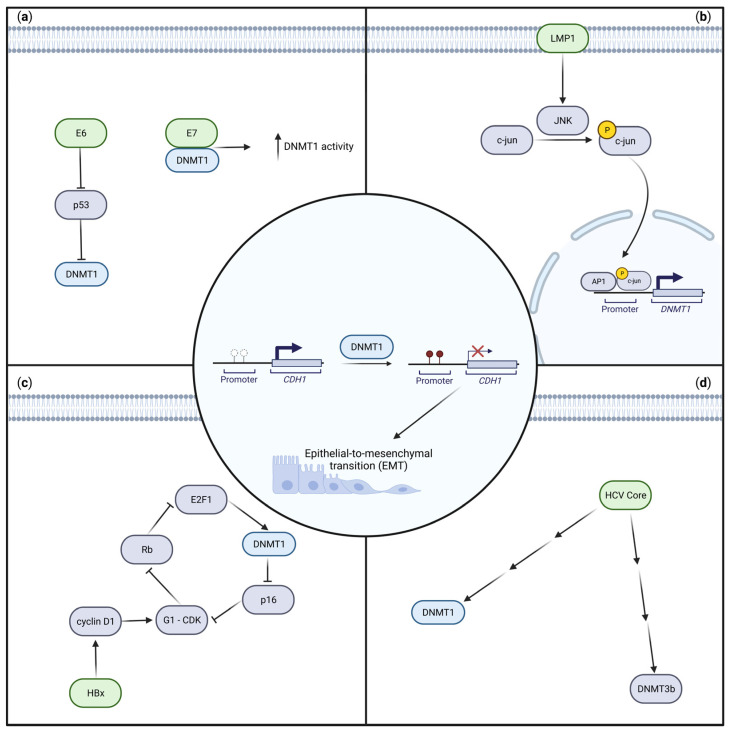
Upregulation of DNA methyltransferase 1 (DNMT1) and subsequent E-cadherin (CDH1) repression by HPV, EBV, HBV, and HCV oncoproteins. Repression of E-cadherin, encoded by the *CDH1* gene, is mediated by upregulation of DNMT1 by viral oncoproteins and subsequent hypermethylation of the *CDH1* promoter in multiple virally driven malignancies (red cross indicates silencing of transcription). This is a key step in epithelial-to-mesenchymal transition (EMT), which is linked to invasion and metastasis. Viral oncoproteins are shown in green, DNMT1 in blue, and all other cellular genes/proteins in purple. The final unifying mechanism of DNMT1 methylating the *CDH1* promoter is shown in the centre. (**a**) Two HPV oncoproteins, E6 and E7, upregulate *DNMT1* expression. E6 may upregulate DNMT1 by repressing p53 [88], whereas E7 binds directly to DNMT1 to stimulate its activity [73,74]. (**b**) The EBV oncoprotein LMP1 activates c-jun NH2-terminal kinase (JNK), which leads JNK to phosphorylate c-Jun, which binds to AP-1 and ultimately the *DNMT1* promoter to drive its expression [43]. (**c**) The HBx protein activates cyclin D1, subsequently upregulating DNMT1 downstream [75]. (**d**) The HCV core protein upregulates DNMT1, as well as DNMT3B, but the exact mechanisms are unknown [78,89]. Adapted from [43,73,74,75,78,88,89]. Created with BioRender.com (accessed on 28 May 2023).

E-cadherin, encoded by the *CDH1* gene, is one of many proteins facilitating cell–cell adhesion, is lost as part of EMT [87], and is a common target of oncogenic viruses [41,43,53,73,74,75,76,78,88]. Mechanistically, HPV, EBV, HBV, and HCV downregulate E-cadherin by oncoprotein-mediated upregulation of DNMT1 and subsequent hypermethylation of the *CDH1* promoter (Figure 2). This repression is achieved by the HPV, EBV, HBV, and HCV oncoproteins E6 [53,88] and E7 [73,74], latent membrane protein 1 (LMP1) [43], HBV X protein (HBx) [75,76], and HCV core protein [78,89], respectively.

Although each of these viral oncoproteins facilitate increased DNMT1 activity, the mechanistic details vary between viral oncoproteins. For instance, in the case of HPV-driven cervical cancer, E7 binds directly to DNMT1 to promote its activity [73,74], whereas increased *E6* expression indirectly leads to higher *DNMT1* expression [53,88], potentially via an E6-mediated pathway promoting p53 repression [88]. Other virally driven mechanisms also activate pathways leading to DNMT1 upregulation [43,75,89]. For instance, in EBV-driven nasopharyngeal carcinoma (NPC) cell lines, LMP1 activates the c-jun N-terminal kinase (JNK) pathway, thereby leading to DNMT1 upregulation [43]. The HBV oncoprotein HBx upregulates cyclin D1, ultimately inhibiting pRB and leading to E2F1 activation of *DNMT1* transcription [75]. HCV core protein also upregulates DNMT1, as well as DNMT3B, but the exact mechanisms are unknown [78,89].

Viral proteins have multiple ways of deregulating EMT. For instance, the *SFRP* genes encode Wnt antagonists, and their repression by promoter hypermethylation leads to constitutive Wnt signalling in a wide variety of cancers [90,91]. Notably, this silencing has been linked to EMT in a number of virally driven cancers [92,93,94]. Inhibition of SFRPs derepresses *SLUG*, *TWIST*, and *SNAIL* expression, thereby downregulating E-cadherin [92]. In hepatocellular carcinoma (HCC), *SFRP* genes are frequently inactivated, and this is also observed in premalignancy, with *SFRP2* predicted to be inactivated by promoter hypermethylation in 33% and 42% of HBV-driven and HCV-driven hepatitis samples, respectively [95]. Interestingly, the hypermethylation of the *SFRP1* gene promoter is associated with expression of HBx or HCV core protein [93,94]. In HNSCCs, *SFRP4* promoter methylation is significantly associated with HPV positivity and not HPV-negative HNSCCs, which are usually driven by alcohol or smoking, suggesting that HPV plays a role in this hypermethylation [42]. Although correlative evidence points to HPV, HBV, and HCV playing a role in SFRP promoter hypermethylation, the mechanisms driving *SFRP* promoter hypermethylation in virally driven malignancies are currently unknown.

### 4.2. Escape from Apoptosis

Apoptosis, or the controlled induction of cell death, is necessary for a multitude of cellular processes [96]. Apoptosis requires tight regulation since escape from molecular safeguards can propel a cell towards an immortal cancerous state. Apoptosis can be triggered either intrinsically via mitochondrial outer membrane permeabilization (MOMP) [97] or extrinsically via death receptor signalling [98]. Ultimately, both pathways culminate in the induction of the caspase cascade, which results in the systematic breakdown of cellular components and eventually cell death [96].

The BCL-2 family governs entry into the intrinsic apoptotic pathway, which includes both pro-apoptotic (i.e., BIM, PUMA, BAX, BAK, and Noxa) and anti-apoptotic (i.e., BCL-2, BCL-XL, BCL-W, BFL1, and MCL1) proteins. Essentially, intrinsic apoptotic induction relies upon a molecular tipping point, where the expression of pro-apoptotic genes overcomes the expression of anti-apoptotic genes [96]. Oncogenic viruses can promote escape from apoptosis by suppressing the expression of pro-apoptotic BCL-2 members, sometimes using epigenetic mechanisms [68,69,70,81,99,100,101,102]. For instance, EBV downregulates the pro-apoptotic protein BIM through both transcript degradation by viral miRNAs arising from both BamHI fragment A right transcript (BART) clusters [100] and a more complex mechanism involving both histone modification and promoter methylation [68,69,70]. As part of this latter mechanism, the EBV proteins EBNA3A and EBNA3C recruit the polycomb repressive complex 2 (PRC2) to the *BIM* promoter, resulting in the deposition of the repressive H3K27me3 mark and subsequent DNA methylation, thus inactivating BIM [68,69,70].

Another similar BIM silencing mechanism has been proposed for an HTLV-1 oncoprotein, HBZ, in the case of adult T-cell leukaemia (ATL) [81]. By nuclear sequestering of FOX03a, HBZ may decrease histone acetylation at the *BIM* promoter by further binding to HATs, p300 and CBP, thereby promoting their dissociation. As EZH2, the catalytic component of the PRC2, is upregulated in ATL, this opens the door to repression via the deposition of H3K27me3 at the *BIM* promoter [81].

More indirectly, *PRDM14* promoter methylation, mediated by HPV infection, is linked to silencing of two pro-apoptotic proteins, PUMA and Noxa, in cervical and oral cancer cell lines [102]. Notably, PUMA is also downregulated by the EBV miRNA miR-BART5 in NPC and GC, representing yet another hurdle to apoptosis induction [99].

### 4.3. Altered Cellular Metabolism

Metabolic reprogramming is a hallmark of cancer [19]. For instance, alterations in metabolic enzymes can lead to drastic changes to epigenetic phenotypes [103]. A classic example in malignancy is neomorphic mutation in *IDH-1* or *IDH-2*, which precipitates the formation of the oncometabolite 2-hydroxyglutarate, ultimately resulting in the CIMP by impaired function of epigenetic modifiers [34,104,105]. Altered cellular metabolism in malignancy is now understood to be highly complex, heterogeneous, and context-specific [106].

Uncovering how oncogenic viruses epigenetically exploit cellular energetics in cancers offers important clues into cancer pathophysiology. Oncogenic viruses frequently promote the Warburg effect (i.e., aerobic glycolysis) by pushing infected cells towards glycolysis [67,71,72,107,108]. For instance, the EBV oncoprotein LMP1 can bind to PARP1 and co-activate *HIF-1α* by the addition of the activating H3K27ac mark to the promoters of *HIF-1α* target genes, ultimately leading to a glycolytic switch in B cell lymphoma cell-line models [107]. Furthermore, LMP1 also upregulates DNMT1 and facilitates its mitochondrial localization, resulting in repression of oxidative phosphorylation through hypermethylation of the mitochondrial DNA (mtDNA) D-loop region [67].

In hypoxic conditions, infection with KSHV results in HIF stabilization and subsequently promotes glycolysis [108]. Specifically, KSHV-induced metabolic rewiring *in vitro* is dependent on a KSHV oncoprotein, viral G-protein coupled receptor (vGPCR), and is associated with large changes in the transcriptomes of KSHV+ cell lines. Interestingly, evidence for large-scale transcriptional reprogramming in KSHV correlates with lower expression of *DNMT3A* and *DNMT3B*, suggesting that DNA methylation may facilitate this metabolic switch to glycolysis [108].

Glycolytic activation in cervical cancer is partially achieved by the HPV oncoprotein E6, which downregulates miR-34a, resulting in the upregulation of the miR-34a target and glycolytic enzyme lactate dehydrogenase A (LDHA) [71]. The HPV oncoprotein E7 also utilizes LDHA to drive oncogenesis [72]. The generation of reactive oxygen species (ROS) in the nucleus by E7 triggers both LDHA nuclear translocation and LDHA to adopt a new enzymatic role to produce the antioxidant α-hydroxybutyrate (α-HB). The generation of α-HB serves to offset the damaging effects of ROS and to activate Wnt signalling by increasing H3K79me3, further promoting cervical cancer cell proliferation [72].

### 4.4. Angiogenesis

Angiogenesis, the process of new blood vessel growth, allows a tumour to sustain its oxygen and nutrient requirements by increasing perfusion from surrounding vasculature [109]. Angiogenesis is now understood to be reciprocally regulated by tumours and their surrounding tumour microenvironments (TMEs), as pro- and anti-angiogenic signals flow between stromal and malignant cells [109]. There is evidence for epigenetic regulation of this process, including examples in virally driven malignancies [51,83,110,111,112].

The spectrum of epigenetic mechanisms used to promote angiogenesis in virally driven malignancies is perhaps best epitomized by KSHV infection in Kaposi’s sarcoma (KS) and primary effusion lymphoma (PEL) [51,83,110,111,112]. Notably, KSHV downregulates the TGF-β signalling pathway using epigenetic mechanisms involving miRNAs, DNA methylation, and histone deacetylation, all of which promote angiogenesis [82,83,110,111,112].

Infection with KSHV silences expression of the anti-angiogenic genes *THBS1*, *TGFBR2*, and *SMAD5* with viral miRNAs [110,111,112]. In one of these studies, to control for the effects of KSHV viral oncoproteins, 10 KSHV miRNAs were ectopically expressed in two different KSHV cell lines, which identified 83 human genes as potential targets of these miRNAs [110]. The study focused on one of these gene targets, *THBS1*, as it had previously been described as downregulated in KS samples [110,113]. Further experiments found evidence that four miRNAs were predicted as the main drivers of THBS1 downregulation at both the mRNA and protein levels and that this downregulation correlated with reduced TGF-β signalling [110]. Other genes involved in the TGF-β signalling pathway were later found to be targets of KSHV viral miRNAs. For example, variants of KSHV miR-K10 targeted the *TGFBR2* transcript [111], and miR-K12-11 was found to downregulate *SMAD5* expression [112]. More indirectly, KSHV viral oncoproteins, vFLIP and vCyclin, upregulated host miR-17-92, which in turn targeted *SMAD2*, resulting in inhibition of TGF-β signalling [83]. Another KSHV oncoprotein, latency associated nuclear antigen (LANA), epigenetically silenced expression of *TGFBR2* by binding to its promoter, which subsequently resulted in DNA methylation and H4 deacetylation [82].

KSHV viral oncoproteins can also promote angiogenesis through mechanisms independent of TGF-β signalling [51]. For instance, KSHV oncoprotein viral interferon regulatory factor 1 (vIRF1) upregulates a host circRNA, circARFGEF, which in turn acts as a sponge for miR-125a-3p, ultimately increasing the expression of the pro-angiogenic protein, GLRX3. In this study, circARFGEF was found to bind to and degrade miR-125a-3p, which targets *GLRX3*. Consequently, *GLRX3* expression was de-repressed [51].

### 4.5. Inflammation

As part of the healing response, inflammatory signals recruit a multitude of immune cells to sites of tissue damage or infection. Yet, inflammation is also a fuel for cancer initiation and progression, as it facilitates genomic instability, angiogenesis, metastasis, oxidative stress, and DNA damage [114]. Predictably, persistent viral infection is a driver of inflammation and a contributor to malignancy initiation. Such is the case in HCC, as chronic hepatitis caused by HBV or HCV infection promotes a pro-tumorigenic environment [13].

Viral epigenetic mechanisms can modulate the expression of pro- or anti-inflammatory molecules as part of carcinogenesis [77,115,116,117]. Viral infection can promote inflammation to mediate cancer initiation by remodelling parts of the host epigenome. For example, as part of HCC pathogenesis, parts of the HBV genome may integrate into the host genome [77]. A result of this integration can be the formation of HBx-long interspersed element 1 (HBx-LINE1), an HBV-human chimeric transcript. HBx-LINE-1 can promote liver inflammation, by acting as a sponge for miR-122. The resulting lack of protective miR-122 promotes liver inflammation in mouse models as part of HCC pathogenesis [77].

The cancer-promoting effects of inflammation often depend on the degree of inflammation, with higher levels of inflammation not always translating to increased cancer cell proliferation [114,115]. For instance, in NPC, low levels of the EBV pro-inflammatory oncoprotein LMP1 promote cell growth, but too much LMP1 has an inhibitory effect on growth. To tune LMP1 levels to support NPC oncogenesis, EBV miR-BART cluster 1 downregulates LMP1 [115].

Virally mediated inflammation may also modulate the function of immune cells in the TME. In terms of epigenetic mechanisms, overexpression of EBV miR-BART11 was found to downregulate FOXP1 in tumour-associated macrophages (TAMs), and this was correlated with increased release of inflammatory cytokines and cancer cell proliferation in NPC cell lines [116].

### 4.6. Generation of Genomic Instability

In most malignancies, genomic instability provides the necessary fuel for the accelerated acquisition of mutations and chromosomal rearrangements [118,119]. Errors in DNA replication, most commonly C>T transitions at methylated CpGs, as well as mutations caused by exogenous and endogenous carcinogens, are common consequences of genomic instability. More recently, different chromatin conformations have been linked to variable mutation rates [119]. Heterochromatic regions also tend to be inaccessible to DNA repair machinery, and this facilitates larger-scale chromosomal rearrangements [119].

Importantly, oncogenic viruses promote genomic instability using diverse molecular mechanisms [80,120,121]. One such mechanism with a well-documented role in genomic instability is the integration of the viral genome into the host genome. This phenomenon has been extensively studied in HPV-driven cancers and HBV-driven HCC [122,123,124]. For instance, the integration of HBV in HCC can drastically reorganize the genome, resulting in widespread gene expression dysregulation and potentially leading to loss of *TP53* via an integration-associated translocation [123]. Intriguingly, in terms of effects on the host epigenome, HBV integrations are over-represented in CpG islands, suggesting that integration aberrantly affects DNA methylation in HCC [120].

Viral oncoproteins can also promote genomic instability via the activation of oncogenic host miRNAs [80]. In the context of ATL, the HTLV-1 oncoprotein HBZ induces the expression of miR17 and miR21 in CD4+ T cells. Since these miRNAs normally downregulate OBFC2A, a DNA-damaging gene, downregulating their expression leads to increased genomic instability [80].

## 5. Epigenetic Biomarkers and Therapeutic Targets of Virally Driven Cancers

Although vaccines are available to combat infection for a subset of oncogenic viruses [1,2], the rates of virally driven cancers remain high in some regions [2], indicating a need for additional preventative and therapeutic strategies to combat virally driven cancer development. Epigenetic changes spurred by viral infection may be used in the future to aid in early diagnosis, prognosis prediction, and inform the use of epigenetic therapies for the treatment of virally driven malignancies [8,54,79].

Translating virally mediated epigenetic changes to the clinic is often not readily practicable. However, there is great potential for innovation given the recent surge of publications in the field. Here, we will provide an overview of biomarkers that may inform diagnosis or prognosis in the future. We will also highlight the use of epigenetic therapeutics in pre-clinical models.

### 5.1. Diagnostic and Prognostic Biomarkers

Oncogenic viruses can modulate host methylation patterns at a genome-wide scale and these patterns can be used to distinguish between viral and non-viral tumours [75,125,126,127,128]. For instance, HNSCCs can be separated by HPV status based on methylation differences within the host genome [125,129]. In particular, HPV-positive HNSCCs tend to have higher methylation levels in genic and LINE1 regions compared to HPV-negative HNSCCs [129]. Longer survival in HPV-positive HNSCCs compared to HPV-negative HNSCCs is well-established [130], and HPV-specific methylation signatures in HNSCC also correlate with improved patient outcomes [131,132]. Similarly, KSHV-driven PEL samples have distinct methylation patterns compared to their KSHV-negative counterparts [126], as do MCPyV-positive compared to MCPyV-negative MCC cell lines [133]. Infection with EBV is associated with a CIMP in NPC and GC, resulting in aberrant gene expression patterns and oncogenic pathway dysregulation [127,134]. Importantly, high-CIMP GC tumours driven by EBV infection are associated with improved patient prognosis compared to lower-CIMP tumours [135].

DNA methylation of oncogenic viral genomes also frequently varies between non-malignant and malignant tissues [136,137,138,139,140]. For instance, the methylation status of select CpG sites in the HPV genome can distinguish between different stages of cervical cancer progression [136], and specific CpG sites are hypermethylated in HBV-driven HCC compared to non-malignant inflamed liver tissues [137]. More recently, circulating viral DNA methylation status has been shown to distinguish non-malignant infection from virally driven malignancy [139,140,141,142]. These so-called liquid biopsies may allow for non-invasive diagnosis of virally driven cancers in the future. In EBV-driven NPC, such tests have relied on the premise that latent EBV genomes are highly methylated and associated with malignancy, whereas virions of EBV tend to be unmethylated and not associated with malignancy [140].

Liquid biopsies can also carry viral miRNAs, which may inform diagnosis or prognosis of virally driven cancers [143]. The EBV miRNA BHRF1-1 is significantly elevated in the plasma of individuals with chronic lymphocytic leukaemia (CLL), highlighting its potential as a diagnostic biomarker [144]. In terms of prognostic biomarkers, miR-BART7 and miR-BART13 were found to be specific to EBV-driven NPC and correlated with advanced-stage disease [145]. In sum, epigenetic biomarkers hold great potential to inform diagnosis and prognosis using non-invasive techniques.

### 5.2. Epigenetic Therapeutic Targets

**Figure 3:**
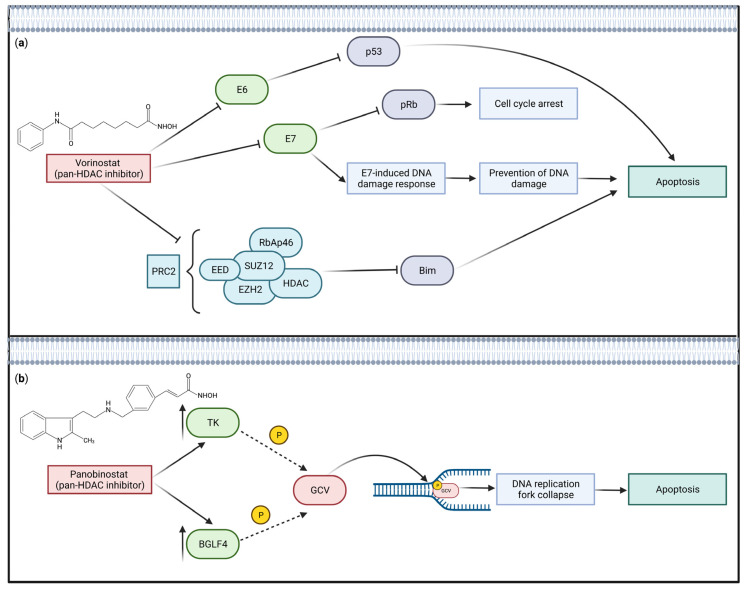
Histone deacetylase (HDAC) inhibitors can promote virally driven cancer cell death. HDAC inhibitors have multiple therapeutic effects in pre-clinical models of virally driven malignancies. Example mechanisms are illustrated here: (**a**) Downregulation of viral oncoprotein expression and apoptosis initiation—vorinostat, a pan-HDAC inhibitor, downregulates *E6* and *E7* expression in HPV18, leading to upregulation of BIM and apoptosis induction [146]. (**b**) Sensitization of cancer cells to antivirals and lytic reactivation—HDAC inhibitors sensitize EBV-infected lymphoma cells to ganciclovir, an antiviral, by induction of the lytic cycle [147]. Viral oncoproteins are shown in green, cellular genes/proteins in purple, complex proteins in blue, and pharmaceutical agents in red. Labels and cellular processes are shown in boxes. EED—embryonic ectoderm development; SUZ12—suppressor of zeste 12 protein homolog; RbAp36—retinoblastoma suppressor-associated protein 46; pRb—retinoblastoma protein; GCV—ganciclovir. Adapted from [146,147]. Created with BioRender.com (accessed on 28 May 2023).

Much interest surrounds the therapeutic use of histone deacetylase (HDAC) inhibitors for virally driven cancers, as there is evidence that HDAC inhibitors downregulate the expression of viral oncoproteins [146], sensitize cancer cells to antivirals [147], activate apoptosis [146,148,149], and spur lytic reactivation [147,150,151] (Figure 3). In HPV18-infected primary human keratinocytes, treatment with vorinostat, a pan-HDAC inhibitor, downregulates expression of *E6* and *E7*, ultimately leading to upregulation of the pro-apoptotic protein BIM and apoptosis induction [146]. The authors of the latter study suggested multiple potential mechanisms for the ability of vorinostat to downregulate E6 and E7, including vorinostat’s ability to limit cells transitioning into G2, the stage wherein HPV replicates [146]. In EBV-infected lymphoma cells, HDAC inhibitors sensitize cells to the antiviral ganciclovir by inducing lytic reactivation, because ganciclovir targets EBV genes not expressed in latency [147]. Similarly, HDAC inhibitor treatment induces KSHV reactivation and results in PEL cell death in cell-line models [150]. Interestingly, HDAC inhibitors may even have clinical utility to prevent HCV-induced iron accumulation in the liver, a risk factor for HCC development. Expression of hepcidin prevents toxic iron accumulation in the liver and HCV infection produces ROS, which induces histone hypoacetylation, thereby blocking access of TFs to the hepcidin promoter [152].

Another target for epigenetic therapeutics in HPV- and HTLV-1-driven malignancies is EZH2 [54,79]. The oncoproteins Tax and E7, from HTLV-1 and HPV, respectively, hijack expression of EZH2, resulting in increased deposition of the repressive H3K27me3 mark [54,79]. In the cervical cancer context, this hijacking mediates apoptosis escape and cell cycle progression [54]. Furthermore, overexpression of *EZH2* is correlated with inferior patient outcomes in HPV-positive oropharyngeal squamous cell carcinoma (OPSCC), also making EZH2 a particularly attractive epigenetic therapeutic target in this cancer type [153]. Pre-clinical models show promise for epigenetic therapies in virally driven malignancies, though exactly how this will translate to the clinic remains to be seen.

## 6. Conclusions and Future Directions

Research into epigenetic mechanisms co-opted by oncogenic viruses has taught us much about cancer pathology, viral mechanisms, and how the two are intertwined, and has revealed many new research avenues to explore.

One noticeable gap in the literature is the scarcity of predictive biomarkers for virally driven cancers. Such research is highly translatable to the clinical setting, since stratifying patients to therapies based on the presence or absence of biomarkers can improve survival or reduce treatment-associated morbidity [154]. This gap is particularly evident in HPV-driven HNSCC. Although HPV status is itself a biomarker of improved prognosis in HNSCC [130], treatments have evolved little in the past few decades and treatment de-escalation for HPV-positive patients remains a priority in ongoing clinical trials [155]. Given that virally driven cancers are well-characterized genomically (i.e., through consortia, such as The Cancer Genome Atlas (TCGA) and the Pan-Cancer Analysis of Whole Genomes (PCAWG) [156]), it is reasonable to hypothesize that additional biomarkers can be discovered in the epigenomes of these cancers.

Tying together viral and host epigenome changes and linking these to the hallmarks of cancer continues to both pose a challenge and present an opportunity for further discovery. Furthermore, the pool of druggable targets is theoretically larger in virally driven cancers, given the presence of both host and viral antigens, heightening the potential for identifying novel targets. Clearly, a better understanding of epigenome dysregulation in cancer may lie at the intersection of the oncology and virology fields.

## Figures and Tables

**Table 2:** Epigenetic mechanisms mediated by viral oncoproteins and their impacts on the host epigenome.

Oncogenic Virus	Oncoprotein	Mechanism	Impact on Host Epigenome	Impact on Cancer Pathology	Refs.
EBV	− LMP1	− Direct interaction with *DNMT1* promoter, driving its overexpression	− Hypermethylation of numerous promoters, including *CDH1*	− EMT, metabolic reprogramming	[43,67]
	− EBNA3A and EBNA3C	− Recruit PRC2 to *BIM* promoter	− Repression of *BIM* transcription via H3K27me3 and DNA methylation	− Escape from apoptosis	[68,69,70]
HPVs (high-risk)	− E6	− Downregulation of miR-34a	− Upregulation of LDHA	− Metabolic reprogramming	[71]
	− E7	− Neomorphic LDHA generation via ROS production	− Production of α-HB increasing H3K79me3 and activating Wnt signalling	− Metabolic reprogramming, increased cell proliferation	[72]
		− Promotes *EZH2* expression in cervical cancer	− Increased deposition of repressive H3K27me3 mark	− Escape from apoptosis and increased cell proliferation	[54]
	− E6 and E7	− Upregulation of *DNMT1* expression	− Hypermethylation of numerous promoters, including *CDH1*	− EMT	[53,73,74]
HBV	− HBx	− Upregulation of DNMT1 via p16 promoter hypermethylation	− Hypermethylation of numerous promoters, including *CDH1*	− EMT	[75,76]
		− HBx-LINE1 acts as sponge for miR-122	− Lack of miR-122 expression	− Inflammation	[77]
HCV	− HCV core protein	− Activates transcription of *DNMT1* and *DNMT3B*	− Hypermethylation of numerous promoters, including *CDH1*	− EMT	[78]
HTLV-1	− HBZ	− Sequesters FOX03a and binds to p300/CBP to promote their dissociation from the *BIM* promoter in ATL	− Repression of BIM via deposition of H3K27me3 by PRC2 (EZH2 is upregulated in ATL)	− Escape from apoptosis	[79]
		− Increases miR17 and miR21 expression, resulting in downregulation of OBFC2A	− Increased expression of oncogenic miRNAs	− Genomic instability	[80]
	− Tax	− Promotes EZH2 activity in ATL	− Increased deposition of repressive H3K27me3 mark	− Escape from apoptosis	[81]
KSHV	− LANA	− Binds to TβRII promoter, resulting in DNA methylation and H4 deacetylation	− Inhibition of TGF-β signalling	− Angiogenesis	[82]
	− vFLIP and vCyclin	− Upregulates miR-17-92, which targets *SMAD2*	− Inhibition of TGF-β signalling	− Angiogenesis	[83]
	− vIRF1	− Upregulates circARFGEF, which acts as a sponge for miR-125a-3p	− Increases expression of *GLRX3*	− Angiogenesis	[51]
MCPyV	− Small T antigen	− Binds to L-MYC to recruit EP400 chromatin remodelling complex	− Transcriptional regulation of multiple genes	− Cell viability and stemness	[84]

HBx—HBV X protein; DNMT1—DNA methyltransferase; PRC2–Polycomb repressive complex 2; LDHA—lactate dehydrogenase A; CBP—CREB binding factor; EZH2—enhancer of zeste homolog 2; BIM—Bcl-2 like 11; α-HB—α-hydroxybutyrate. Note that not all references are referred to in the main text.

## Data Availability

Not applicable.
